# Antihypertensive Drug Use: A Racial Decomposition Analysis Based on the Largest Alzheimer’s Disease Registry Data

**DOI:** 10.1093/geroni/igaf122.1838

**Published:** 2025-12-31

**Authors:** Kevin Lu, Xiangxiang Jiang, Gang Lv

**Affiliations:** University of South Carolina, Columbia, South Carolina, United States; University of South Carolina, Columbia, South Carolina, United States; Chinese PLA General Hospital, Beijing, Beijing, China (People’s Republic)

## Abstract

**Background:**

Mortality from Alzheimer’s Disease and Related Dementias (ADRD) has more than doubled in the past two decades, with South Carolina leading the nation in crude ADRD death rates. African Americans face a disproportionately higher risk of ADRD, with widening racial disparities, partly driven by comorbid conditions like hypertension. However, limited research has quantified the contribution of racial disparities to healthcare costs and utilization. This study addresses this gap by analyzing racial differences in healthcare expenditures, utilization, and antihypertensive medication (AHM) use among South Carolina Medicaid beneficiaries with ADRD, leveraging data from the South Carolina Alzheimer’s Disease (AD) Registry.

**Methods:**

A longitudinal analysis was conducted using SC Medicaid claims data linked to the SC AD Registry, the nation’s oldest and most comprehensive statewide ADRD database. The study included ADRD patients aged 65 and older (2005–2021). Healthcare costs were adjusted to 2024 dollars. The Blinder-Oaxaca decomposition method was used to quantify racial disparities in inpatient, outpatient, emergency room, and medication expenditures.

**Results:**

Among 7,865 ADRD Medicaid beneficiaries, racial differences accounted for 23.52%–34.37% of disparities in inpatient, outpatient, and medication expenditures (P < 0.001). Racial disparities explained 28.95% (P < 0.001) of AHM use differences.

**Conclusion:**

Racial disparities significantly contribute to variations in healthcare costs, utilization, and medication use among ADRD patients. Addressing these disparities is critical for ensuring equitable access to care and improving outcomes.

